# The thematic role of extracellular loop of VraG in activation of the membrane sensor GraS in a cystic fibrosis MRSA strain differs in nuance from the CA-MRSA strain JE2

**DOI:** 10.1371/journal.pone.0270393

**Published:** 2022-06-23

**Authors:** Junho Cho, William F. C. Rigby, Ambrose L. Cheung

**Affiliations:** 1 Department of Microbiology and Immunology, Geisel School of Medicine, Dartmouth College, Hanover, New Hampshire, United States of America; 2 Department of Medicine, Geisel School of Medicine, Dartmouth-Hitchcock Medical Center, Lebanon, New Hampshire, United States of America; Laurentian University, CANADA

## Abstract

Patients with cystic fibrosis (CF) often suffer recurrent bronchial bacterial infections that lead to deterioration of lung function over time. The infections in CF patients are often due to *S*. *aureus* and *P*. *aeruginosa* that colonize the airways. Significantly, methicillin-resistant *S*. *aureus* (MRSA) makes it challenging for treatment in CF patients due to its feature of multiple antibiotic resistance. In bronchial airways, cationic antimicrobial peptides are often present in mucosa cells, neutrophils, and macrophages that interfere with bacterial proliferation. The major mechanism for resistance to the bactericidal activity of cationic peptides in *S*. *aureus* is mediated by the GraRS two-component system that activates expression of MprF and DltABCD to increase surface positive charge to repel interactions with cationic peptides. We recently found that VraG, a membrane permease component of the VraFG efflux pumps, harbors a long 200-residue extracellular loop (EL) that utilizes K380 to interact with the negatively charged 9-residue extracellular loop of the membrane sensor GraS to control *mprF* expression in a community-acquired MRSA strain JE2. In this study, we extended this observation to a CF MRSA strain CF32A1 where we affirmed that the EL loop of VraG controls GraS-mediated signal transduction; however, in contrast to community acquired MRSA strain JE2, the CF MRSA strain CF32A1 requires both K380 and K388 in the EL of VraG to properly modulate signal transduction mediated by GraS. This effect was not attributable to the several single nucleotide polymorphisms that exist between VraG and GraS in the two MRSA strains.

## Introduction

Methicillin-resistant *Staphylococcus aureus* (MRSA), along with *Pseudomonas aeruginosa*, has been considered as causative pathogens of chronic pulmonary infections in cystic fibrosis (CF) patients [1–4]. In the case of *S*. *aureus*, the microorganism is prevalently spread from the CF respiratory tracts to the lung tissues. The formation of small colony variants (SCVs) as well as increased antibiotic resistance renders the lung infections almost intractable to treatment [5, 6].

A microscopic study of lung specimens in CF patients showed that *S*. *aureus* cells were primarily found in the mucus of respiratory tracts and not directly on lung epithelial cells [7]. Some groups also reported its adherence onto bronchial trees [8, 9]. Using an *ex vivo* pig lung model, it was observed that *S*. *aureus* resided in lung tissue-associated biofilm and preferentially aggregated in artificial sputum medium surrounding lung tissues [10]. Together, these data support the notion that colonization of CF lung by *S*. *aureus* results in serious pulmonary infections, leading to residual lung damage which facilitates repeat infections.

Human cationic antimicrobial peptides, also called human defense peptides (HDP), play critical roles in eliminating bacterial pathogens in healthy and CF airways [11]. Human defense peptides (a.k.a. defensins), comprising mainly of α and β-defensins that are produced in innate immune cells and epithelial cells, are cationic cysteine-rich amphipathic peptides with broad-spectrum antimicrobial activities [12]. For instance, histatins comprising histidine-rich peptides target MRSA and various oral pathogens [13]. Cathelicidin LL-37, expressed in epithelial cells and human neutrophils, expresses bactericidal activities, stimulates immune responses by releasing cytokine and chemokine and inhibits biofilm formation [14]. These HDPs can disrupt membrane integrity by inserting into bacterial membrane followed by oligomerization and pore formation, leading to permeabilization. To evade these cationic antimicrobial peptides, *S*. *aureus* utilizes a two-component systems (TCS), called GraRS [15] to increase the membrane surface positive charge (via enhanced MprF and DltABCD expression) and activates an efflux pump (VraFG encoding an ATPase and membrane permease, respectively) thought to expel the cationic peptides away from bacterial cells [16, 17]. In addition, the GraRS TCS is thought to be essential for survival in phagolysosomes of neutrophils with an acidic environment [18–20]. The acidic pH is thought to activate the GraRS regulon to confer resistance to antimicrobial effectors, including antimicrobial peptides and oxidative stress [21].

Molecular analysis indicated that the single 9-residue extracellular loop (EL) of the membrane sensor GraS in *S*. *aureus* is critical to its activation by auto-phosphorylation followed by a phosphor-relay to secondary activation of the response regulator GraR [22]. Recently, we found that the 200-residue EL of VraG, a permease encoded by the *vraFG* efflux pump system, played an important regulatory role in GraS-mediated signal transduction in a community-acquired MRSA strain JE2 [23]. More specifically, deleting the EL of VraG stimulated activation of GraS, even in the absence of cationic peptides, showing increased surface positive charge accompanied by enhanced survivability when exposed to LL-37 and neutrophils [23]. Mutagenesis study revealed that positively charged lysine residue at position 380 in the EL of VraG played a key role in inhibiting GraS sensing in JE2, possibly by masking the EL of GraS. To verify if MRSA clinical isolates from CF patients also inherit these features for HDPs sensing, we conducted this current study which showed that the EL of VraG in a CF MRSA strain CF32A1 appears to inhibit GraS-mediating sensing in a manner similar to JE2. However, unlike JE2, both K380 and K388 in the EL of VraG in CF32A1 are required to modulate the activation of GraS. Thus, there is a similar thematic trend in inhibition of GraS-mediated sensing by EL of VraG in *S*. *aureus*. However, nuance exists because the specific residues involved in this inhibition may differ between strains.

## Results

### The extracellular loop of VraG regulates GraRS TCS in MRSA strain CF32A1

We have obtained a clinical isolate (CF32A1) from a cystic fibrosis patient at Dartmouth Hitchcock Medical Center. Based on the position of EL within VraG (residues 309–508) and the finding that VraG of CF32A1 differs from JE2 with a conservation substitution (T231I), we constructed the following mutants: Δ*graS*, Δ*vraG*, ΔEL *vraG*, *vraG* with a double mutation at K380A, K388A, *vraG* K380A, *vraG* K388A and *vraG* with a triple mutation at K327A, K331A, K343A (randomly chosen as a negative control for lysine mutations), using allelic replacement with pMADx, a recombinant pMAD constructed for this study (Table 1). The complemented mutants were constructed by replacement of the mutation with the native gene.

**Table 1:** Strains and plasmids.

Strains and plasmids	Features	Reference
CF32A1	A cystic fibrosis MRSA isolate; erm^r^	This study
pMAD	A plasmid used for allelic replacement in *s*. *aureus*; erm^r^ for *S*. *aureus*, amp^r^ for *E*. *coli*	[24]
pMADx	pMAD in which erm^r^ gene was replaced by cm^r^ gene; cm^r^ for *S*. *aureus*, amp^r^ for *E*. *coli*	This study
pMADx::Δ*graS*	pMADx with DNA fragment corresponding to upstream and downstream of *graS*	This study
pMADx::Δ*vraG*	pMADx with DNA fragment corresponding to upstream and downstream of *vraG*	This study
pMADx::ΔEL *vraG*	pMADx with DNA fragment corresponding to upstream and downstream of EL *vraG*	This study
pMADx::*vraG* K327A, K331A, K343A	pMADx with *vraG* gene with the K327A, K331A, K343A mutation	This study
pMADx::*vraG* K380A, K388A	pMADx with *vraG* gene harboring K380A, K388A mutation	This study
pMADx::*vraG* K380A	pMADx with *vraG* gene harboring K380A mutation	This study
pMADx::*vraG* K388A	pMADx with *vraG* gene harboringK388A mutation	This study
pMADx::*vraG* I231T	pMADx with *vraG* gene harboring I231T mutation	This study
pMADx::*vraG* ΔEL, I231T	pMADx with *vraG* DNA fragment corresponding to upstream and downstream of EL of *vraG* accompanied by I231T mutation	This study
pMADx::*vraG* K380A, K388A, I231T	pMADx with *vraG* gene harboring K380A, K388A, I231T mutations	This study
pMADx::*vraG* K380A, I231T	pMADx with *vraG* gene harboring K380A, I231T mutations	This study
pMADx::*graS* L26F, I59L	pMADx with *graS* gene harboring L26F, I59L mutation	This study
pMADx::*graS*	pMADx with the native *graS*	This study
pMADx::*vraG*	pMADx with the native *vraG*	This study
IM08B	An *E*. *coli* enabling plasmid transformation into clonal complex 8 (CC8) *S*. *aureus*	[25]
pALC1484	A plasmid with promoterless GFPuvr gene; cm^r^ for *S*. *aureus*, amp^r^ for *E*. *coli*	[26]
pALC1484::*mprF*	pALC1484 with *mprF* promoter	[23]
Δ*graS*	CF32A1 with *graS* deletion	This study
Δ*vraG*	CF32A1 with *vraG* deletion	This study
ΔEL *vraG*	CF32A1 with the extracellular loop deletion of *vraG*	This study
*vraG* K327A, K331A, K343A	CF32A1 with lysine to alanine mutation in residues 327, 331, and 343 of VraG	This study
*vraG* K380A, K388A	CF32A1 with lysine to alanine mutation in residues 380 and 388 of VraG	This study
*vraG* K380A	CF32A1 with lysine to alanine mutation in residue 380 of VraG	This study
*vraG* K388A	CF32A1 with lysine to alanine mutation in residue 388 of VraG	This study
*vraG* I231T	CF32A1 with isoleucine to threonine mutation in residue 231 of VraG	This study
*vraG* ΔEL, I231T	ΔEL *vraG* with isoleucine to threonine mutation of residue 231	This study
*vraG* K380A, K388A, I231T	*vraG* with K380A, K388A mutations and isoleucine to threonine mutation in residue 231	This study
*vraG* K380A, I231T	*vraG* with K380A mutation and isoleucine to threonine mutation in residue 231 of VraG	This study
*vraG* K380A, K388A, *graS* L26F, I59L	*vraG* K380A, K388A with a double mutation, L26F and I59L, of *graS*	This study
*vraG* K380A *graS* I26F, I59L	*vraG* K380A with a double mutation, L26F and I59L, of *graS*	This study
Δ*graS* complement	Δ*graS* complemented with native *graS*	This study
Δ*vraG* complement	Δ*vraG* complemented with native *vraG*	This study
ΔEL *vraG* complement	ΔEL *vraG* complemented with native *vraG*	This study
*vraG* K380A, K388A complement	*vraG* K380A, K388A mutant complemented with native *vraG*	This study
*vraG* K380A complement	*vraG* K380A mutant complemented with native *vraG*	This study
CF32A1 pALC1484::*mprF*	CF32A1 with pALC1484::*mprF* promoter	This study
Δ*graS* pALC1484::*mprF*	Δ*graS* with pALC1484::*mprF* promoter	This study
Δ*vraG* pALC1484::*mprF*	Δ*vraG* with pALC1484::*mprF* promoter	This study
ΔEL *vraG* pALC1484::*mprF*	ΔEL *vraG* with pALC1484::*mprF* promoter	This study
*vraG* K327A, K331A, K343A pALC1484::*mprF*	*vraG* K327A, K331A, K343A mutant with pALC1484::*mprF* promoter	This study
*vraG* K380A, K388A pALC1484::*mprF*	*vraG* K380A, K388A mutant with pALC1484::*mprF* promoter	This study
*vraG* K380A pALC1484::*mprF*	*vraG* K380A mutant with pALC1484::*mprF* promoter	This study
*vraG* K388A pALC1484::*mprF*	*vraG* K388A mutant with pALC1484::*mprF* promoter	This study
*vraG* I231T pALC1484::*mprF*	*vraG* I231T mutant with pALC1484::*mprF* promoter	This study
*vraG* ΔEL, I231T pALC1484::*mprF*	*vraG* ΔEL, I231Tmutant with pALC1484::*mprF* promoter	This study
*vraG* K380A, K388A, I231T pALC1484::*mprF*	*vraG* K380A, K388A, I231T mutant with pALC1484::*mprF* promoter	This study
*vraG* K380A, I231T pALC1484::*mprF*	*vraG* K380A, I231T mutant with pALC1484::*mprF* promoter	This study
Δ*graS* complement pALC1484::*mprF*	Δ*graS* complement with pALC1484::*mprF* promoter	This study
Δ*vraG* complement pALC1484::*mprF*	Δ*vraG* complement with pALC1484::*mprF* promoter	This study
ΔEL *vraG* complement pALC1484::*mprF*	ΔEL *vraG* complement with pALC1484::*mprF* promoter	This study
*vraG* K380A, K388A, *graS* L26F, I59L pALC1484::*mprF*	*vraG* with K380A, K388A mutation combined with *graS* with L26F, I59L mutation harboring pALC1484::*mprF* promoter	This study
*vraG* K380A *graS* I26F, I59L pALC1484::*mprF*	*vraG* with K380A mutation combined with *graS* L26F, I59L mutation harboring pALC1484::*mprF* promoter	This study
*vraG* K380A, K388A complement pALC1484::*mprF*	*vraG* K380A, K388A complement with pALC1484::*mprF* promoter	This study
*vraG* K380A complement pALC1484::*mprF*	*vraG* K380A complement with pALC1484::*mprF* promoter	This study

We first determined the sensitivity of the mutants to the cationic peptide polymyxin B (PMB) (Table 2). Corresponding to the previous study [23], the Δ*graS* and Δ*vraG* mutants in CF32A1 exhibited increased susceptibility to PMB, with the MIC declining ~8 folds, compared to the wild type. While the MIC of the ΔEL *vraG* mutant was higher than the *vraG* and *graS* mutants, it remained at the level of the parent at ~64 μg/ml. Unexpectedly, the *vraG* K380A mutants also had MICs of ~ 64 μg/ml, similar to the wild type while *vraG* with the double mutation at K380A and K388A exhibited an MIC of ~32 μg/ml. In contrast to MRSA strain JE2 where the K380A mutation increased PMB susceptibility by four-folds [23], we surmised that the roles of EL and, in particular, the lysine 380 residue (K380) in VraG in CF32A1 MRSA for HDP sensing might be altered in CF32A1 as compared to JE2.

**Table 2:** MIC of PMB for vraG mutants in CF32A1.

Strains	MIC of PMB (μg/ml)
CF32A1 (Wild type)	**64**–128
Δ*graS*	**8**–16
Δ*vraG*	**8**–16
ΔEL *vraG*	**64**–128
*vraG* K327, 331, 343A	**64**–128
*vraG* K380A, K388A	**32**–64
*vraG* K380A	**64**–128
*vraG* K388A	**64**–128
Δ*graS* complement	**64**–128
Δ*vraG* complement	**64**–128
ΔEL *vraG* complement	**64**–128
*vraG* K380A, K388A complement	**64**–128
*vraG* K380A complement	**64**–128

The bold numbers indicate median values from at least three biological replicates.

**Fig 1:**
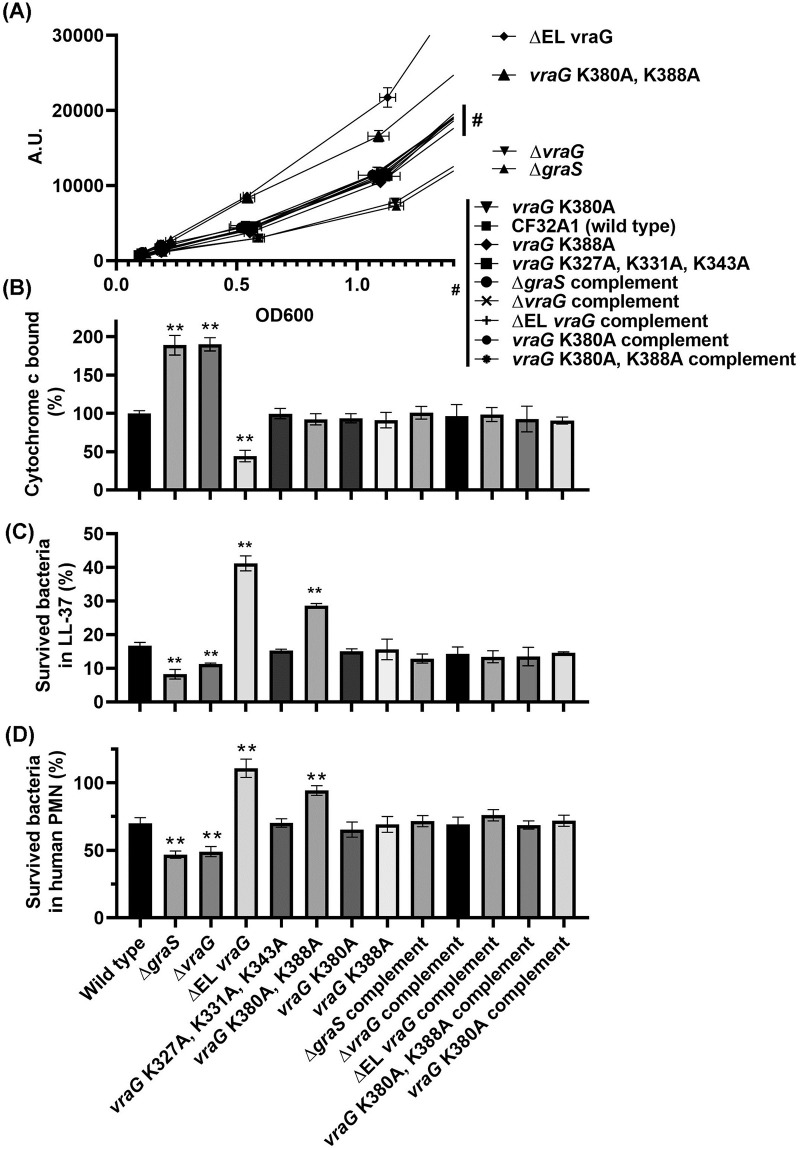
Effects of EL *vraG* mutants on GraRS two-component system. **(A)** GFPuvr expression driven by the *mprF* promoter among various mutants. The fluorescence of GFPuvr (arbitrary unit, A.U. in y-axis) and OD600 in x-axis were monitored every hour for 5 hours. The strains for overlapped lines drawn by nine samples (|#) are separately listed below. **(B)** cytochrome c binding assay. Cells grown to the mid-log phase were treated with 0.25 mg/ml cytochrome c, and the percentages of cytochrome c bound were normalized vs. the wild type cells set at 100%. All the measurements were collected from at least three biological replicates. **(C)** LL-37 2hr killing assay and **(D)** PMN killing assay were independently performed at least three times. The representative data set were displayed with technical replicates. The asterisks (**) indicate p < 0.01.

To investigate the effects of the *vraG* mutations on GraS-mediating sensing of HDPs, we transformed the mutants with a plasmid harboring *mprF* promoter fused to the *gfp*_*uvr*_ reporter gene (Table 1). Since *mprF* expression is highly induced by GraS-mediated signal transduction [15], GFPuvr expression stimulated by activation of the *mprF* promoter enabled us to estimate the level of activation of GraS (Fig 1A). The parental strain (CF32A1) had a constitutive *mprF* expression with slope values 9434–10496 with 95% confidence intervals in linear regress analysis, which were calculated from fluorescent intensity (arbitral unit, A.U.), with the OD600 ranging from inoculation to exponential phase (OD600 of ~1). The ΔEL *vraG* mutant (slope values 19151–21443) revealed two-fold higher *mprF* expression than the wild type, whereas the Δ*graS* (slope values 5709–6709) and Δ*vraG* (slope values 5931–7281) mutants exhibited a much lower capacity for *mprF* expression vs. the parent. Interestingly, the *vraG* with the double mutation at K380A and K388A (slope value 15439–17000) displayed much lower *mprF* expression than the ΔEL *vraG* mutant but higher than the wild type. Unexpectedly, the vraG K380A mutant (slope values 9857–11508) did not make conspicuous changes in *mprF* expression, dissimilar to the previous study [23]. As controls, the *vraG* mutant with the triple mutation at K327A, K331A, K343A and the complemented strains of Δ*graS*, Δ*vraG*, ΔEL *vraG*, *vraG* K380/388A, *vraG* K380A displayed *mprF* expression equivalent to the wild type. These data indicate that the EL of *vraG* interferes with the GraS-mediated sensing; however, the double mutation K380A and K388A in *vraG* can in part overcome the “veiled” effect of the EL of VraG for lifting a coordinated regulation of GraS in CF32A1.

MprF is an enzyme that links lysine residues to membrane phospholipids to increase the surface positive charge. To ascertain if the enhanced *mprF* expression in the ΔEL *vraG* and *vraG* with the double mutation at K380A and K388A mutants are associated with elevated surface positive charge, we measured the cytochrome c binding of each mutant as a percentage of the wild type set at 100% (Fig 1B). The Δ*graS* and Δ*vraG* mutants bound more cytochrome c at ~1.9 times more than the wild type, corresponding to reduced positive charge on the cell membrane surface. In contrast, the ΔEL *vraG* mutant reduced the binding of cytochrome c by ~50%, as compared to the wild type. Unexpectedly, the *vraG* with the double mutation at K380A and K388A did not exhibit a change in cell surface charge, with binding of cytochrome c similar to the wild type; this finding could be due to a lack of high sensitivity in this assay which requires a moderate amount of change in surface charge to increase binding of cytochrome c. To affirm if hyperexpression of *mprF* in the ΔEL *vraG* mutant and the *vraG* mutant with the double mutation at K380A and K388A leads to discernable and relevant phenotypes, we evaluated bacterial survivability upon 2 hr. exposure to LL-37, a cationic HDP found in neutrophils and human keratinocytes. As shown in Fig 1C, the ΔEL *vraG* mutant survived more than two-fold better in LL-37 than the parental strain. The *vraG* with the double mutation at K380A and K388A also displayed increased survivability higher than the parent but lower than the ΔEL *vraG* mutant, in parallel to *mprF* expression (Fig 1A). Predictably, the Δ*graS* and Δ*vraG* mutants had reduced survival in LL-37, ~ 50% lower than the parent. The increased resistance of ΔEL *vraG* and *vraG* with the double mutation at K380A and K388A to LL-37 appeared to be correlate with survival upon exposure to human PMN (Fig 1D). More specifically, the ΔEL *vraG* mutant survived much better than the parent, exceeding the loaded inoculum (> 100% survivability) while the *vraG* with the double mutation at K380A and K388A showed higher survivability in human PMNs than the parent, but lower than the ΔEL *vraG* mutant. As expected, survival of the Δ*graS* and Δ*vraG* mutants was similar and less than the parent and complemented mutants.

### The variant T231I in EL of VraG in CF MRSA strain CF32A1 does not affect GraS-mediated *mprF* expression

**Fig 2:**
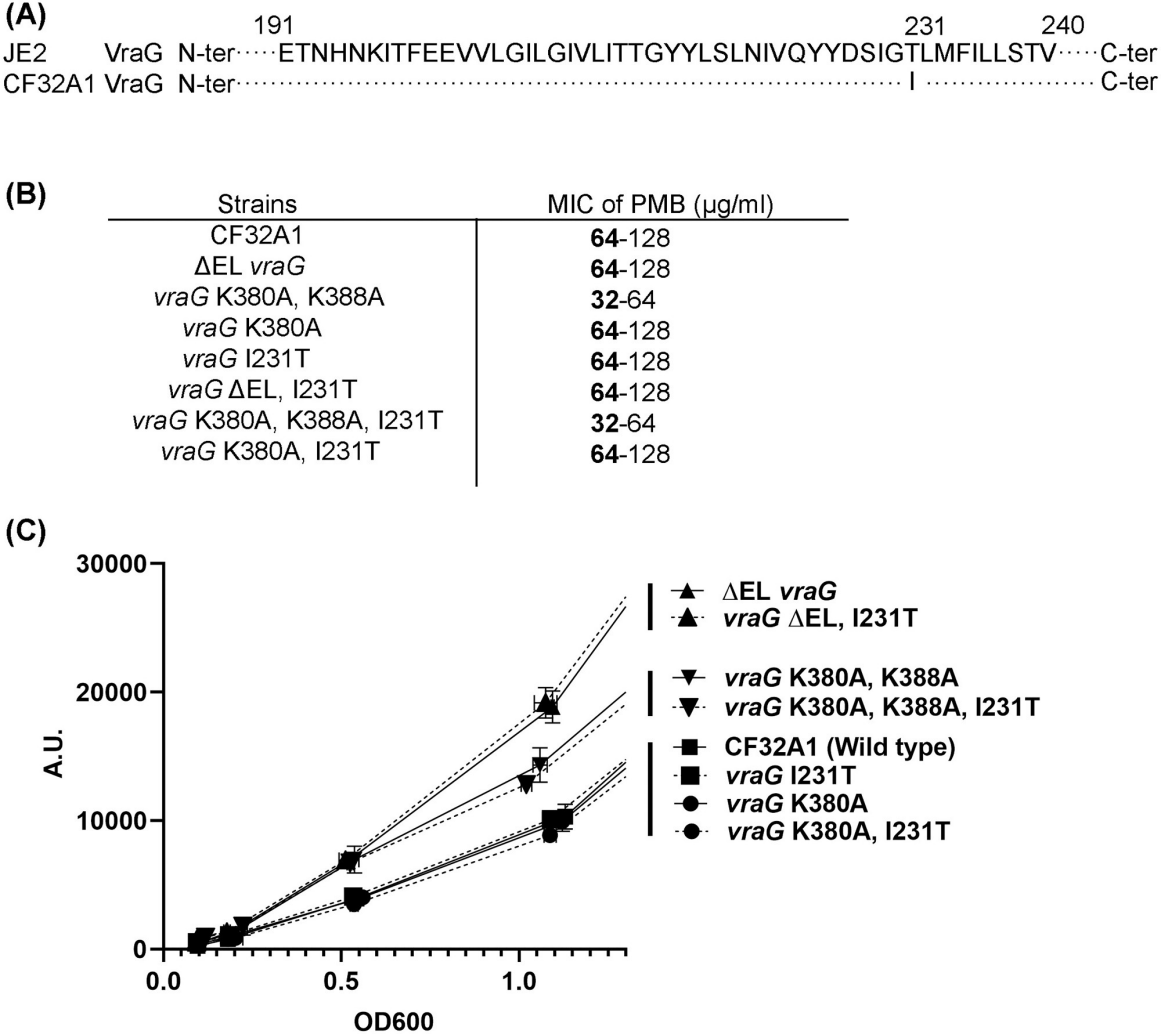
Missense mutation I231 in VraG. **(A)** Comparison of VraG sequence in CF32A1 vs. JE2. **(B)** MIC of PMB for vraG mutants. Median values from three biological replicates are marked in bold. **(C)** The expression of *mprF* promoter fused with a GFPuvr reporter in assorted strains. The dashed lines indicate the mutants with *vraG* I231T.

To understand the discrepancies of GraS-mediated activation between *vraG* K380A mutants in JE2 and CF32A1, we aligned the amino acid sequence of VraG between the two strains and found that VraG in CF32A1 has an isoleucine at position 231 (Ile231), compared to threonine at identical position in JE2 (Thr231) (Fig 2A). According to TOPCONS prediction of VraG, I231 is located in the 6^th^ transmembrane segment in VraG, away from the 200-residue EL loop [27]. The VraG variant with T231I substitution in CF32A1 is frequently found in community-acquired MRSA CN1 and various clinical isolates (S3 Table). To check if residue I231 in VraG plays any roles in modulating GraS-mediated signal transduction, we constructed additional mutations in CF32A1 including *vraG* I231T, ΔEL *vraG* with I231T, *vraG* with the triple mutation at K380A, K388A, I231T, and *vraG* with the double mutation at K380A and I231T. We found that the I231T mutation in the ΔEL *vraG* mutant of CF32A1 did not affect susceptibility to PMB, displaying MICs of ~ 64 μg/ml similar to their parent (Fig 2B). Importantly, *vraG* carrying the double mutation at K380A and I231T had MIC similar to the *vraG* K380A mutant, indicating that the I231T mutation in CF32A1 did not alter MIC to PMB. Likewise, the *vraG* I231T mutant exhibited *mprF* expression indistinguishable from the isogenic parent, similar to the single K380A and the double K380A and I231T mutants (Fig 2C).

**Fig 3:**
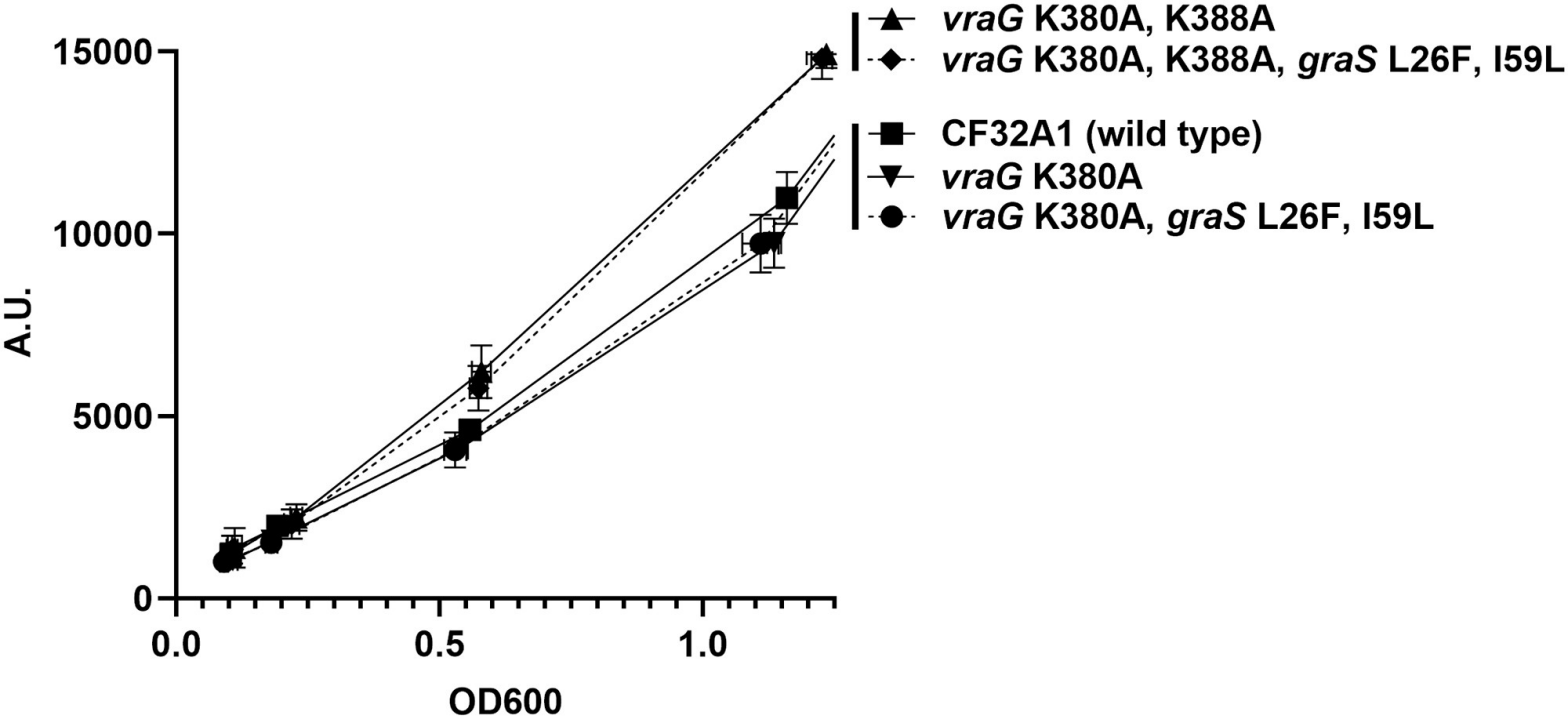
The effects of GraS L26F, I59L on *mprF* expression in VraG K380A mutant. The expression of *mprF* promoter fused with a GFPuvr reporter were monitored from the wild type strain and four mutants. All the values were calculated from three biological replicates. The dashed lines indicate the mutants with *graS* L26F, I59L.

In examining GraS in MRSA CF32A1 and JE2, we found that GraS has two variants at residues 26 (leucine) and 59 (isoleucine) in CF32A1, whereas these residues are phenylalanine (F) and leucine (L) at positions 26 and 59, respectively, in the JE2 strain. To ensure that these variants did not impact GraS-mediated signal transduction (e.g., *mprF* expression), we constructed two CF32A1 mutants by changing L26 and I59 residues of GraS to F26 and L59 in wild type and *vraG* mutants including the double K380A, K388A mutant and the single K380A mutant. However, these variants harboring the L26F and I59L mutations did not lead to a significant change in *mprF* expression from their isogenic parents or mutants (Fig 3). From these results, we concluded that the GraS L26, I59 variant as well as VraG I231 variant in CF32A1 are irrelevant to GraS-mediated sensing activity; however, additional factors in CF32A1 that may affect the interaction between ELs of VraG and GraS for *mprF* activation cannot be entirely ruled out.

## Discussion

The GraS-VraFG system bears a resemblance to the BceRS-BceAB TCS responsible for bacitracin resistance in *B*. *subtilis* [28]. In this system, activation of BceR by BceS via a phosphorelay induces expression of the BceAB efflux pump, a homolog of VraFG in *S*. *aureus*. Interestingly, BceS sensor activity degenerates, and the EL of the permease BceB substitutes the function to recognize bacitracin [29, 30]. In contrast, GraS, as a homolog of BceS, keeps the recognition function to a nine-residue EL of GraS for HDPs sensing [31]. Although the sensor ability of EL of VraG has been postulated to exist [22], physical evidence to support this scheme has not been uncovered until recently.

In a recent study, we showed that the EL of VraG in MRSA strain JE2, which encompasses ~200 residues, represses GraS-mediated sensing as assessed by *mprF* expression [23]. Removal or a K380A mutation in the EL of VraG leads to enhanced GraS-mediated signaling even in the absence of cationic antimicrobial peptides such as PMB. Collective evidence suggested that the EL of VraG most likely interacts with the EL sensing loop of GraS as a coordinated sensor to interfere with sensing activation and removal of the EL activates it. In this study, we reaffirmed the role of EL of VraG in GraS-mediated sensing in CF MRSA strain CF32A1 by comparing features of various *vraG* mutants in CF32A1 and JE2 in GraS-mediated sensing of cationic peptides (S2 Table). In JE2, the ΔEL *vraG*, K380A, and K380A/K388A mutants were accompanied by a transient or temporary change in *mprF* expression and membrane surface charge, showing enhanced survival in the short-term bactericidal activities with LL-37 and 1 hr. killing assay by human PMNs [23]. These findings cannot be explained by a defective permease lacking efflux activity alone. In contrast, the ΔEL *vraG*, *vraG* K380A/K388A, and *vraG* K380A mutans from CF32A1 exhibited little change on PMB sensitivity vs. the parent while the Δ*vraG* mutant impacted significantly on PMB susceptibility (8 vs. 64 μg/ml in the parent), consistent with a defective efflux pump and sensing as assessed by *mprF* expression.

The most noticeable result here was that a single K380A or K388A mutation in the EL of VraG in CF32A1 did not affect sensing of GraS but a double mutation with K380A and K388A enhanced GraS-mediated sensing, similar to the ΔEL *vraG* mutant as reflected by elevated *mprF* expression and increased survivability in LL-37 and PMN vs. the parent (Fig 1 and S2 Table). This is in sharp contrast to JE2 where a single K380A mutation alone was sufficient to alter GraS-mediated signal transduction, though not as effective as the ΔEL mutant. Despite our finding here, it is plausible that the K380A mutation alone in CF32A1 might affect GraS activation to a much lesser extent, but our assays are not sensitive enough to detect the subtle changes vs. the parent. Nevertheless, three common themes can emerge by comparing these two strains on GraS activation via VraG. First, the interaction between EL of GraS and VraG probably involves charge-charge interaction, with the three negatively charged aspartic residues in the 9-residue EL of GraS interacting with the positively charged lysine residues (K380 and/or K388) on the EL of VraG. Second, there are strains variation in terms of the lysine residues (K380 or K380/388) required for this interaction. Third, the GraS sensing as mediated by VraG does not rely on the efflux activity of the VraG permease.

One variation in residue 231 in VraG which becomes an isoleucine in CF32A1 instead of a threonine (as found in JE2) did not impact on MIC, *mprF* expression, 2 hr. killing with LL37, and human PMN killing assays, indicating residue 231 in VraG, which is not part of the EL, plays no role in GraS-mediated activation. Additionally, two variations (L26 and I59) in GraS of CF32A1, which are located at the transmembrane segments, did not affect *mprF* expression as well. Thus, the single nucleotide polymorphism at position 231 in VraG and positions 26 and 59 in GraS did not impact GraS-mediated sensing in MRSA strain CF32A1 and community acquired MRSA strain JE2.

The EL of VraG, as a long extracellular loop of a membrane permease component of an efflux pump, would be predicted to be able to contact the peptidoglycan layer and intermingle with numerous loops of membrane proteins including the 9-residue EL (35-DYDFPIDSL-43) of the GraS membrane sensor which comprises three critical aspartic acid residues (D35, 37, 41) for interaction with lysine residues (K380 and K380/388) in EL of VraG. We theorize that disruption of this ionic interaction unleashes activation of GraS. Despite this common theme, there are nuances in this ionic interaction between strains that K380 of VraG is important for JE2 whereas both K380 and K388 are critical for CF32A1.

Collectively, we reconfirmed that the EL of VraG is involved in HDPs sensing by controlling GraS signal transduction and lysine residues (K380 or K380/388) in the EL of VraG play a vital role in GraS activation. As the double mutants (K380A & K388A) in both CF32A1 and JE2 were not as effective as the ΔEL in activating GraS-mediated signal transduction, we surmise that the bulky EL structure or charged residues K380 and 388 on the exposed surface of the EL might partially hinder activation of GraS. In addition, the ΔEL might change membrane fluidity in VraG as it resides between 7^th^ and 8^th^ transmembrane segments. Whether this interaction promotes GraS dimerization (promoting phosphorylation and signaling) is under current study.

## Methods

### Strain and plasmid construction

The clinical staphylococcus aureus strain CF32A1 was a gift from Dr. Hogan at Dartmouth college. Since CF31A1 is resistant to erythromycin, we constructed a new pMAD plasmid (denoted as pMADx) for allelic replacement of genomic DNA by replacing the *erm*^r^ gene in pMAD with *cm*^r^ gene in pSK236 (Table 1, S1 File), utilizing Gibson assembly (NEB, NEBuilder HIFI DNA assembly Master Mix). All the mutants listed in Table 1 were prepared with pMADx, following the procedures as described in a previously reported article [24] except for the antibiotic selection using 10 μg/ml of chloramphenicol. In short, target genes of interest were PCR-amplified with primers listed on S1 Table and then ligated into pMADx, followed by transformation into *E*. *coli* strain IM08B for proper methylation prior to transformation into *S*. *aureus*. The isolated pMADx vector with a target gene from *E*. *coli* was transformed into CF32A1 with selection on an X-gal TSA plate (Goldbio cat# X4281C) with 10 μg/ml cm. Light blue colonies were selected after inducing a crossover event at 43°C. The double crossover/plasmid curing events were carried out at 30°C. The desired mutants were chosen from white colonies in an X-gal TSA plate and confirmed by DNA sequencing.

### MIC of PMB

The strains from overnight culture in TSB were diluted (1:1000) and seeded and regrown in 5 ml CA-MHB (BD Difco^™^ Mueller Hinton Broth) at 37°C. At a mid-log phase (OD600 ~ 0.7), the cells were diluted to make a final concentration of 1 X 10^6^ CFU/ml as indicated in the CLSI standard [32]. 100 μl of the diluted cells were mixed with 100 μl of a serial dilution of PMB (RPI cat# P40160, Sigma cat# P1004) as described in a CLSI method. After 16–18 hours of incubation at 37°C, we screened the lowest concentration of PMB at which cell growth was completely inhibited in 96 well plates.

### GFPuvr expression

We transformed the CF32A1 mutants with *mprF* promoter fused to GPF_uvr_ reporter in plasmid pALC1484 (pALC1484::*mprF* promoter) as described [23]. Notably, the *mprF* promoter sequence in CF32A1 was identical to the one in JE2, allowing interchangeability of the plasmid between strains. The cells were grown in TSB (BD Bacto^™^ Tryptic soy broth) with 10 μg/ml cm overnight and diluted in 10 ml CA-MHB (1:100 dilution). The fluorescence of GPF_uvr_ from the mutants was measured at excitation λ = 487 and emission λ = 511 with intervals of 1 hour, using Tecan (Infinite M1000pro, Tecan Inc.). The data were collected and analyzed from three biological measurements.

### Cytochrome c binding assay

The strains in 10 ml of CA-MHB were grown to OD600 of 0.7. The cells were washed and resuspended in 700 μl of MOPS. The resuspension was diluted to the final concentration of A_650_ = 3. Cell pellet from 500 μl of the resuspension was mixed with equal volume of 0.25 mg/ml cytochrome c (Sigma cat# C2506) in MOPS. The mixture was incubated with a rotary at room temperature for 10 min and then centrifuged at 5000 x g for 2 min. The supernatant isolated from the mixture was measured at A_530_ to estimate the amount of unbound cytochrome c. The cytochrome c binding percentages of the mutants were normalized from their bound amount on wild type cells. The measurements were done at least three times with two technical replicates.

### LL-37 2 hr. killing assay

The strains were grown to a mid-log phase (OD600 ~ 0.7) in 5 ml of BHI media (BD BBL^™^ Brain Heart Infusion) at 37°C and diluted in 10 mM KH_2_PO_4_ with 1% BHI (PPB buffer) to make a final concentration of 1 X 10^6^ CFU/ml. The diluted samples were mixed with 2.5 μg/ml LL-37 in PPB buffer and then incubated for 2 hrs. at 37°C. The mixtures were serially diluted and spread on TSA plates. The number of colonies grown on the plate was used to calculate each mutant’s survivability against LL-37, compared to each mutant’s inoculum.

### PMN assay

To isolate PMNs, we followed the procedures described in a previous paper with minor modifications [23, 33]. The human blood was collected from volunteers following a protocol endorsed by Committee for the Protection of Human Subjects of the Geisel School of Medicine at Dartmouth. Whole blood was diluted 1:1 in RPMI and centrifuged over Ficoll gradient (GE Healthcare). The erythrocyte pellet was then re-sedimented in 3% dextran (Alfa Aesar MW ca 500,000). PMN rich layer was isolated and treated with RBC lysis buffer (BD Bioscience, BD Pharm Lyse^™^ cat# 555899) to lyse the remaining erythrocytes. The purified PMNs were washed with HBSS (Corning 21-022-CV), and the number of alive PMNs was estimated with trypan blue exclusion after staining with 0.4% trypan blue. To prepare bacterial samples, we re-grew the bacterial cells to OD600 of ~0.8 from overnight cultures and opsonized them with 1% human serum for 30 min. The opsonized cells were diluted to 1 X 10^7^ CFU/ml, mixed with the fresh PMN (MOI ~ 1 or 2), and incubated at 37°C for 1 hr. Triton-X100 at 0.1% was added to lyse the PMN and the mixture spread on TSA. The survived bacterial ratios were calculated from the number of initial inoculum and post-PMN-treated cells. The assay was independently performed at least three times, and a representative result was illustrated in the figure.

## Supporting information

S1 Table Oligonucleotides used in this study. (DOCX)

S2 Table A summary of EL VraG effects on GraRS TCS in CF32A1 vs. JE2. The data for EL vraG effects in JE2 were referred to the previously reported results [23]. The arrows (↑ and ↓) indicate up and down-regulation caused by the mutations. The double arrows (↑↑ and ↓↓) represent noticeable changes in the mutants when compared to ones with single arrows. N.C. indicates no significant change vs. the parental strain.
(DOCX)

S3 Table List of strains including VraG T231I. (DOCX)

S1 File pMADx information. (DNA)
