# Supplementary material for: The thematic role of extracellular loop of VraG in activation of the membrane sensor GraS in a cystic fibrosis MRSA strain differs in nuance from the CA-MRSA strain JE2
Source: PLoS One. 2022 Jun 23;17(6):e0270393. doi: 10.1371/journal.pone.0270393 (PMC9223312; doi:10.1371/journal.pone.0270393)
Supplement: S1 Table — (DOCX) [file pone.0270393.s001.docx]

**S1 Table. Oligonucleotides.**

| IDs for oligonucleotides. | Sequence (5’ to 3’, bold: restriction sites) | Ref. |
| --- | --- | --- |
| pMADx Gibson Assembly F | GATTTGAGCGTAGCGAAAAATCCTATAACCCTCTTTAATTTGGTTATATGAATTTTG | This study |
| pMADx Gibson Assembly R | CTAATGACTGGCTTTTATAAGTTAAGGGATGCATAAACTGCATC | This study |
| Cm Gibson Assembly F | GTTTATGCATCCCTTAACTTATAAAAGCCAGTCATTAGGCCTATC | This study |
| Cm Gibson Assembly R | CCAAATTAAAGAGGGTTATAGGATTTTTCGCTACGCTCAAAT | This study |
| pMADx IDF 1 | GGGTTATTGTCTCATGAGCG | This study |
| pMADx IDF 2 | GTCGTTTGTTGGTTCAAATAATGATTAAATATC | This study |
| pMADx IDF 3 | GACATAAATCATGAGGAAACATATAAAAATTATG | This study |
| pMADx IDF 4 | GAGAGTTTGAAAGAAGTAGTGAATACATG | This study |
| pMADx IDF 5 | GCAAATCAGATGCAACCGGT | This study |
| *graS*_pMADx_Up_F | AAA**GGATCC**GGTGCGACCGCTCGCAG | [23] |
| *graS*_pMADx_Up_R | AAA**CCCGGG**CCAAAATATCCAGTTCATGCG |  |
| *graS*_pMADx_Down_F | AAA**CCCGGG**GAACGCATGTCGGAAGTGAC |  |
| *graS*_pMADx_Down_R | AAA**GGATCC**CAATACTACACTCGTAATTAACG |  |
| *vraG*_pMADx_Up_F | AAA**GGATCC**GGGGACAACTGTCAGATTG |  |
| *vraG*_pMADx_Up_R | AAA**CCCGGG**GGCATAATGTGATAAATTTTGACG |  |
| *vraG*_pMADx_Down_F | AAA**CCCGGG**GCAGTGACGGCTTATAATCAT |  |
| *vraG*_pMADx_Down_R | AAA**GGATCC**CATCATCAATTGCATCACATAATG |  |
| El of *vraG* pMADx Up F | AAAAA**GTCGAC**CAAAGAAATAATACGTGTACAAAGTGT |  |
| El of *vraG* pMADx Up R | AAAAA**CCCGGG**TGCAGTATATTTTATTTCACTTGATAAGG |  |
| El of *vraG* pMADx Down F | AAAAA**CCCGGG**AGCTCAATATCAAGTTTAACCGG |  |
| El of *vraG* pMADx Down R | AAAAA**GGATCC**ATTTCAAAAATGCCTCACAGTAAA |  |
| *graS* complement pMADx F | AAAAAA**CCCGGG**AGTGGAAGTTTAGTGAAAAAATATACAGTTA |  |
| *graS* complement pMADx R | AAAAAA**GGATCC**ATGGCATAATGTGATAAATTTTGACGG |  |
| *vraG* complement pMADx F | AAAAAA**CCCGGG**TGAAACGACGTCTTCAGGTATG |  |
| *vraG* complement pMADx R | AAAAAA**GGATCC**TCTAAAACATCATCAATTGCATCACATAAT |  |
| pMADx Up F for point mutation in EL *vraG* | AAA**CCCGGG**CTTTAACGAGATAATATTTAAAAATTTTCGTC | This study |
| pMADx Up F for short | AAA**CCCGGG**CTTTAACGAG | This study |
| pMADx Down R for point mutation in EL *vraG* | AAA**GGATCC**CTCTAAAAATTGTAAATGGATATAGTTGG | This study |
| pMADx Down R for short | AAA**GGATCC**CTCTAAAAATTGTAAATGG | This study |
| pMADx Up F for point mutation in *graS* | GCCGATTTATTACTTTATACAAGCACC | This study |
| pMADx Down R for point mutation in *graS* | CAAATTTGTCACTTCCGACATGC | This study |
| pMADx Up F for point mutation in *graS* with XmaI | AAAAA**CCCGGG**CCGATTTATTACTTTATACAAGC | This study |
| pMADx Down R for point mutation in *graS* with BamHI | AAAAA**GGATCC**AAATTTGTCACTTCCGACATG | This study |
| *graS* L26F F | GGATATTGTTTTTAAACTTCCTTATGTTAGGCATTAG | This study |
| *graS* L26F R | CTAATGCCTAACATAAGGAAGTTTAAAAACAATATCC | This study |
| *graS* I59L F | GTTTAACAATGATTTTTCTTTTATTGACATATTTTAAAGAAG | This study |
| *graS* I59L R | CTTCTTTAAAATATGTCAATAAAAGAAAAATCATTGTTAAAC | This study |
| *graS*_IDF_F | CGATGAAGGTAGTTTTACCAAAGG | [23] |
| *graS*_IDF_R | GACTTGTGAGCCTTCCTTTA |  |
| *vraG*_IDF_F | GAACGAGGATTTACGTCAACG |  |
| *vraG*_IDF_R | ACTAATAAGCCGACAGCAAGT |  |
| pMADx_IDF_F | GTTACGTTACACATTAACTAGACAG |  |
| pMADx_IDF_R | GAAGAATCATAATGGGGAAGG |  |
| *vraG* K327, 331, 343A_1 | TTAGCTGCTTGTTGGTCTGCAATTGTAACGTCGTGTGGTG |  |
| *vraG* K327, 331, 343A_2 | TGCTTGATTGTTTAATTCACTTGCTAATTGATTAGCTGCTTGTTGGTCTGC |  |
| *vraG* K327, 331, 343A_3 | GCAGACCAACAAGCAGCTAATCAATTAGCAAGTGAATTAAACAATCAAGCA |  |
| *vraG* K327, 331, 343A_4 | GTGAATTAAACAATCAAGCAATTCCTCATTTTTATAATTATAAAGAAGTAATTCATAC |  |
| *vraG* K380, 388A_1 | GTGCCAAATCAGTATTAGGGATGTATGCATCACTAGTAATTGTTACATTGTATGGTTC |  |
| *vraG* K380, 388A_2 | GATGCATACATCCCTAATACTGATTTGGCACGTGGGCAAGCTGATTTATTTG |  |
| *vraG* I231T_F | CAATATTATGATTCTATCGGTACACTTATGTTTATTTTATTG | This study |
| *vraG* I231T_R | CAATAAAATAAACATAAGTGTACCGATAGAATCATAATATTG | This study |
| *vraG* K380A F | CAATTACTAGTGATGCATACATCCCTAATAC | [23] |
| *vraG* K380A R | GTATTAGGGATGTATGCATCACTAGTAATTG |  |
| *vraG* K388A F | CTAATACTGATTTGGCACGTGGGCAAG |  |
| *vraG* K388A R | CTTGCCCACGTGCCAAATCAGTATTAG |  |
| pALC1484 IDF F | GGCGATTAAGTTGGGTAACG |  |
| pALC1484 IDF R | CTGACAGAAAATTTGTGCCC |  |
| *mprF* promoter pALC1484 F | AAAAAA**GAATTC**CAATCGTTTCTATGGTAATGAT |  |
| *mprF* promoter pALC1484 R | AAAAAA**TCTAGA**TAATTATTTCTGTTATAAATCAAAATATATCA |  |

The bold letters indicate restriction sites.

23. Cho J, Costa SK, Wierzbicki RM, Rigby WFC, Cheung AL. The extracellular loop of the membrane permease VraG interacts with GraS to sense cationic antimicrobial peptides in Staphylococcus aureus. PLoS Pathog. 2021 Mar;17(3):e1009338.
