# Supplementary material for: The thematic role of extracellular loop of VraG in activation of the membrane sensor GraS in a cystic fibrosis MRSA strain differs in nuance from the CA-MRSA strain JE2
Source: PLoS One. 2022 Jun 23;17(6):e0270393. doi: 10.1371/journal.pone.0270393 (PMC9223312; doi:10.1371/journal.pone.0270393)
Supplement: S2 Table — The data for EL vraG effects in JE2 were referred to the previously reported results [23]. The arrows (↑ and ↓) indicate up and down-regulation caused by the mutations. The double arrows (↑↑ and ↓↓) represent noticeable changes in the mutants when compared to ones with single arrows. N.C. indicates no significant change vs. the parental strain. (DOCX) [file pone.0270393.s002.docx]

**S2 Table. A summary of EL VraG effects on GraRS TCS in CF32A1 vs. JE2.**

|  | JE2 *vraG* | | | CF32A1 *vraG* | | |
| --- | --- | --- | --- | --- | --- | --- |
|  | ΔEL | K380,388A | K380A | ΔEL | K380,388A | K380A |
| MIC of PMB | ↓ | ↓ | ↓ | N.C. | N.C. | N.C. |
| *mprF* expression | ↑↑ | ↑↑ | ↑ | ↑↑ | ↑ | N.C. |
| Cytochrome c binding | ↓↓ | ↓ | ↓ | ↓ | N.C. | N.C. |
| 2hr killing assay with LL-37 | ↑↑ | ↑ | ↑ | ↑↑ | ↑ | N.C. |
| PMN assay | ↑ | ↑ | ↑ | ↑ | ↑ | N.C. |

The data for EL vraG effects in JE2 were referred to the previously reported results [23]. The arrows (↑ and ↓) indicate up and down-regulation caused by the mutations. The double arrows (↑↑ and ↓↓) represent noticeable changes in the mutants when compared to ones with single arrows. N.C. indicates no significant change vs. the parental strain.

23. Cho J, Costa SK, Wierzbicki RM, Rigby WFC, Cheung AL. The extracellular loop of the membrane permease VraG interacts with GraS to sense cationic antimicrobial peptides in Staphylococcus aureus. PLoS Pathog. 2021 Mar;17(3):e1009338.
