# Supplementary material for: The thematic role of extracellular loop of VraG in activation of the membrane sensor GraS in a cystic fibrosis MRSA strain differs in nuance from the CA-MRSA strain JE2
Source: PLoS One. 2022 Jun 23;17(6):e0270393. doi: 10.1371/journal.pone.0270393 (PMC9223312; doi:10.1371/journal.pone.0270393)
Supplement: S3 Table — (DOCX) [file pone.0270393.s003.docx]

**S3 Table.** **List of strains including VraG T231I**

| Strains |
| --- |
| *Staphylococcus aureus* UP_274 |
| *Staphylococcus aureus* UP_830 |
| *Staphylococcus aureus* UP_883 |
| *Staphylococcus aureus* 6538P |
| *Staphylococcus aureus* ER03364.3 |
| *Staphylococcus aureus* NP66 |
| *Staphylococcus aureus* B9-22D |
| *Staphylococcus aureus* NRRL B-41012 |
| *Staphylococcus aureus* 2030RH1 |
| *Staphylococcus aureus* NCTC4163 |
| *Staphylococcus aureus* NCTC4137 |
| *Staphylococcus aureus* NCTC7121 |
| *Staphylococcus aureus* NCTC8317 |
| *Staphylococcus aureus* ATCC BAA-39 |
| *Staphylococcus aureus* NX-T55 |
| *Staphylococcus aureus* QD-CD9 |
| *Staphylococcus aureus* S57 |
| *Staphylococcus aureus* F17SA003 |
| *Staphylococcus aureus* E16SA093 |
| *Staphylococcus aureus* NCTC10344 |
| *Staphylococcus aureus* NCTC3761 |
| *Staphylococcus aureus* NCTC6136 |
| *Staphylococcus aureus* NCTC9752 |
| *Staphylococcus aureus* AR_0473 |
| *Staphylococcus aureus* AR_0472 |
| *Staphylococcus aureus* MOK042 |
| *Staphylococcus aureus* MOK063 |
| *Staphylococcus aureus* CFSAN018750 |
| *Staphylococcus aureus* SA003 |
| *Staphylococcus aureus* K5 |
| *Staphylococcus aureus* 2148.N |
| *Staphylococcus aureus* K18 |
| *Staphylococcus aureus* K17 |
| *Staphylococcus aureus* FORC_040 |
| *Staphylococcus aureus* BA01611 |
| *Staphylococcus aureus* ATCC 6538 |
| *Staphylococcus aureus* ST20130943 |
| *Staphylococcus aureus* ST20130942 |
| *Staphylococcus aureus* FORC_012 |
| *Staphylococcus aureus* FDA209P |
| *Staphylococcus aureus* TMUS2134 |
| *Staphylococcus aureus* TMUS2126 |
| *Staphylococcus aureus* 2868B2 |
| *Staphylococcus aureus* NAS_AN_016 |
| *Staphylococcus aureus* NAS_AN_023 |
| *Staphylococcus aureus* NAS_AN_136 |
| *Staphylococcus aureus* NAS_AN_149 |
| *Staphylococcus aureus* NAS_AN_175 |
| *Staphylococcus aureus* NAS_AN_181 |
| *Staphylococcus aureus* NAS_OP_056 |
| *Staphylococcus aureus* NAS_OP_107 |
| *Staphylococcus aureus* NAS_AN_047 |
| *Staphylococcus aureus* NAS_AN_152 |
| *Staphylococcus aureus* NAS_AN_250 |
| *Staphylococcus aureus* NAS_AN_265 |
| *Staphylococcus aureus* NAS_OP_026 |
| *Staphylococcus aureus* NAS_OP_163 |
| *Staphylococcus aureus* NT_8 |
| *Staphylococcus aureus* E1185_IV_ST12 |
| *Staphylococcus aureus* ER15889.6A |
| *Staphylococcus aureus* ER15889.5A |
| *Staphylococcus aureus* ER15889.3A |
| *Staphylococcus aureus* HL24830 |
| *Staphylococcus aureus* HL16278 |
| *Staphylococcus aureus* HL18840 |
| *Staphylococcus aureus* CN1 |
| *Staphylococcus aureus* WHC07 |
| *Staphylococcus aureus* CI/BAC/25/13/W |
| *Staphylococcus aureus* SA14+ |
| *Staphylococcus aureus* MEI001 |
| *Staphylococcus sp.* SM9054 |
| *Staphylococcus sp.* SM3655 |
| *Staphylococcus aureus* 014S_SA |
| *Staphylococcus aureus* DG36 |
| *Staphylococcus aureus* ER10617.3 |
| *Staphylococcus aureus* ER10823.3 |
| *Staphylococcus aureus* ER11011.3 |
| *Staphylococcus aureus* pt223 |
| *Staphylococcus aureus* pt252 |
| *Staphylococcus aureus* pt290 |
